# Pulmonary artery air embolism with consequent primary respiratory alkalosis and secondary metabolic alkalosis following ventilation therapy: A case report

**DOI:** 10.1097/MD.0000000000039078

**Published:** 2024-07-26

**Authors:** Nora A. Altorbak, Rayyan A. Daghistani, Hassan R. Al-Omaish, Thamer A. Alsaab, Shahad K. Alhomaiani

**Affiliations:** a Medical Imaging Department, King Abdulaziz Medical City, Ministry of National Guard Health Affairs, Riyadh, Suadi Arabia; b Medical Imaging Department, King Abdulaziz Specialist Hospital, Taif, Saudi Arabia.

**Keywords:** air embolism, alkalosis, angiography, case report, pulmonary artery, ventilation

## Abstract

**Background:**

An air embolism is a rare complication that occurs after air enters blood vessels, causing almost no to mild symptoms in patients. Although uncommon, air embolism can be deadly. Critical care professionals should know the warning signs of air embolism and be prepared to carry out the necessary therapeutic interventions. To reduce morbidity and death, this clinical condition must be identified early. Here we are presenting a case of pulmonary artery air embolism as a consequence of contrast agent injection in a chest computed tomography study.

**Case presentation:**

A 70-year-old male patient were presented with pulmonary artery air embolism as a consequence of contrast agent injection in a chest computed tomography study. The patient experienced worsening respiratory symptoms that necessitated oxygen therapy, which resulted in respiratory alkalosis with secondary metabolic alkalosis. Following removal of the BiLevel positive airway pressure, the patient was switched to a 2-L nasal cannula, and his breathing rate increased to 34 breaths/min. After 8.5 hours of monitoring the patient’s vital signs, the nasal cannula was removed, and the patient began breathing room air on his own. His vital signs then stabilized and arterial blood gas parameters returned to normal. The patient’s condition improved, and he was discharged from the hospital after 9 days. Due to a high level of cytomegalovirus, the discharge prescriptions included valganciclovir film-coated tablets (900 mg, oral BID every 12 hours for 30 days) and apixaban (5 mg BID). The patient was then monitored at the outpatient clinic.

**Conclusion:**

Although rare, an air embolism can cause minor symptoms if it is small in volume or can be fatal if large. After contrast-enhanced radiological studies, physicians should be aware of any signs of respiratory distress or worsening of symptoms in their patients. Additionally, patients should be mindful of the potential complications associated with ventilation therapy.

## 1. Introduction

A pulmonary air embolism, though rare, can be life-threatening. It can occur when air enters the circulatory system through an artery or vein from various sources, such as iatrogenic procedures, including peripheral venous access, endoscopy, tissue biopsy, and angiography, or even following penetrating trauma.^[1–4]^

Pulmonary air embolism occurrences during pneumoradiography, needle biopsy, or angiography have also been documented.^[3]^ Contrast-enhanced computed tomography (CECT), while a valuable diagnostic tool for radiologists, carries a risk of pulmonary air embolism when air is inadvertently injected into the veins too rapidly using an infusion pump.^[5]^ This was documented by Laasir et al^[5]^ in a case of a 40-year-old woman who developed a venous air embolism in the pulmonary artery after injection of a contrast agent.

Here, we describe the case of a patient with a pulmonary artery air embolism due to the rapid, unintentional injection of a small amount of air during CECT. The patient developed tachypnoea and then respiratory alkalosis with secondary metabolic alkalosis following oxygen therapy. Fortunately, the patient’s condition improved, and he was later discharged. Managing physicians should be aware of this issue to provide appropriate care when it arises.

## 2. Case presentation

A 70-year-old male patient who had been previously diagnosed in 2006 with diffuse large B-cell lymphoma, mainly gastric, presented to our emergency department with a 4-day history of fever, cough, shortness of breath, and decreased oral intake. During lymphoma treatment, the patient had undergone 6 cycles of R-CHOP (rituximab, cyclophosphamide, doxorubicin hydrochloride [hydroxydaunorubicin], vincristine sulfate [Oncovin], and prednisone). In November 2023, he underwent a colonoscopy following complaints of abdominal pain, which showed a gastric ulcer due to recurrence. Subsequently, he received 3 cycles of R-GMOX (rituximab, gemcitabine, and oxaliplatin).

The patient was admitted shortly after undergoing chest computed tomography (CT) angiography. His vital signs showed an increased respiratory rate of up to 42 breaths/min within 1 hour following chest CT, and he was initiated on BiLevel positive airway pressure (BiPap) for 4 hours. The primary physicians recommended transferring the patient to the intensive care unit if he was still tachypneic.

The patient was breathing spontaneously on oxygen at 2 L/min via a nasal cannula, and oxygen saturation improved from an initial level of 92% (room air 90%) to 98% to 99%. His Glasgow Coma Scale score was 15 (15/15 is normal), his blood pressure was 145/78 mm Hg (normal reference range, systolic/diastolic BP 120/80 ± 20 mm Hg), heart rate was 85 beats/min (normal reference range, 60–100 beats/minute), respiratory rate was 35/min (normal reference range, 12–18 breath per minute), and body temperature was 37.8 °C (normal reference range: 36.1–37.2 °C). The patient showed no signs of distress and was conversing well on physical examination.

The laboratory tests findings were as follows: white blood cell count, 14.4 × 10^9^/L (reference range, 4–11 × 10^9^/L); hemoglobin level, 113 g/L (reference range, 135–180 g/L); hematocrit value, 0.343 L/L (reference range, 0.42–0.54 L/L); platelet count, 18 × 10^9^/L (reference range, 180–400 × 10^9^/L); and D-dimer value, 7.25 mg/L (normal reference range, 0–0.5 mg/L).

The managing emergency department physician requested CECT angiography to rule out pulmonary artery embolism due to elevated D-dimer levels. The contrast agent (Xenetix 80–100 mL) was administered via an auto-injector into the antecubital vein.

Since the patient was known to have bronchial asthma, he was administered asthma medications before CT, according to hospital protocol.

However, considering the current clinical presentation and the patient’s known history of lymphoma, the managing physician refused to wait and wished to proceed with chest CT immediately to exclude pulmonary embolism after the administration of the asthma medications since the patient did not show any asthma symptoms before the CT scan.

**Figure 1.**
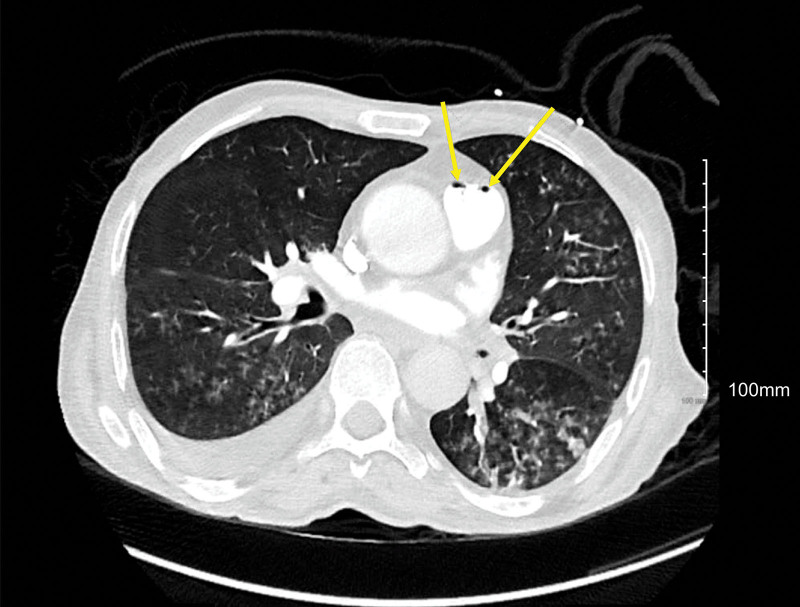
Chest CT scan showing air bubbles in the pulmonary trunk. CT = computed tomography.

**Figure 2.**
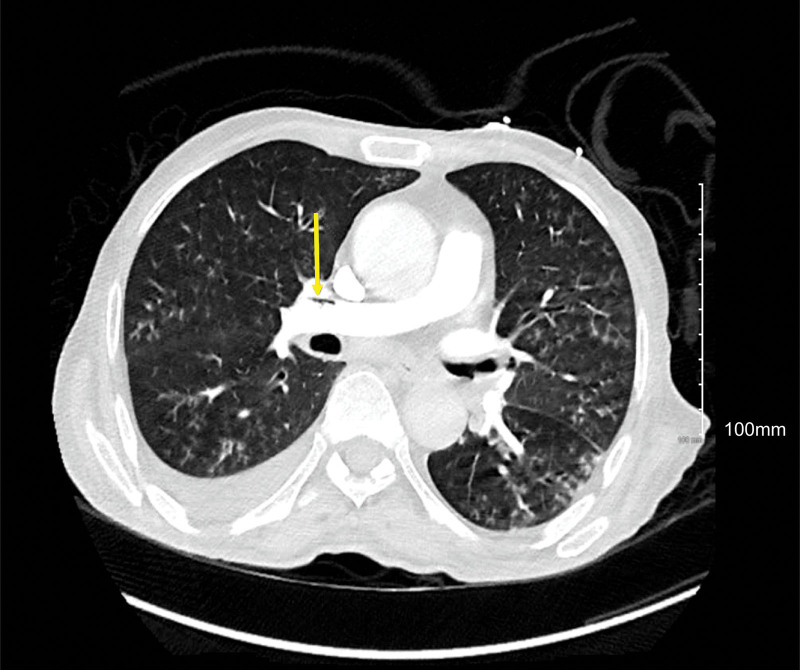
Positive chest CT scan for distal right main pulmonary artery air bubbles. CT = computed tomography.

**Figure 3.**
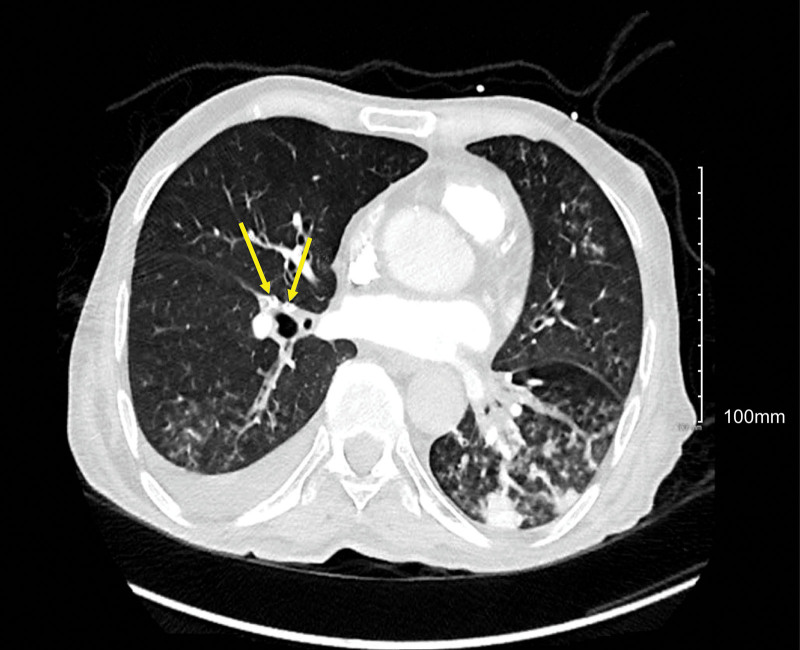
Chest CT scan showing air bubbles in the right lower lobe segmental pulmonary artery. CT = computed tomography.

The chest CT scan was positive for right upper lobe segmental pulmonary artery embolism; it also showed air bubbles in the pulmonary trunk, right main pulmonary artery, and right lower lobe segmental pulmonary artery (Figs. 1–3).

The findings were noted in the radiology reporting system, and the primary nurse was informed as the primary physician did not respond to their pager.

Arterial blood gas (ABG) analysis revealed respiratory alkalosis with secondary metabolic alkalosis after BiPap application. The ABG laboratory test results were as follows: pH, 7.48 (normal reference range, 7.35–7.45); bicarbonate value, 25 (normal reference range, 22–29 mEq/L); carboxyhemoglobin value, 34 (normal reference range, 35–45 mm Hg); partial pressure of carbon dioxide, 34 (normal reference range, 35–45 mm Hg); partial pressure of oxygen, 90 (normal reference range, 75–100); oxygen saturation, 96.9% (high); sodium level, 128 (normal reference range, 136–145 mmol/L); and potassium level, 3.5 (normal reference range, 3.5–5.2 mmol/L). After BiPap removal, the patient was placed on a 2-L nasal cannula, and his respiratory rate improved to 34 breaths/min. The patient’s vital signs were monitored for the following 8.5 hours; he was then weaned off the nasal cannula and was able to spontaneously breath room air. Subsequently, ABG parameters normalized, and his vital signs were stable.

The treatments prescribed to the patient during his hospital stay were as follows: piperacilin/tazobactam (4.5 g IV piggyback bolus injection every 6 hours for 4 days, mixed with sodium chloride 0.9% 100 mL solution); budesonide (500 µg/mL) solution for nebulization (500 µg inhalation via nebulizer [inhaled 2 times/day for 5 days]); ipratropium (250 µg/mL solution for nebulization [inhaled 4 times/day for 5 days]); furosemide injection (Day 1: 40 mg IV push, infusion every 24 hours for 3 days); vancomycin injection (100 mg IV piggyback injection every 6 hours, mixed with dextrose 5% in water [100 mL] for 10 days); meropenem injection (1000 mg piggyback over 15–30 minutes, injected every 8 hours for 4 days); potassium chloride injection (30 meq IV piggyback over 3 hours, mixed with 300 mL of 0.9% sodium chloride solution, once]; and magnesium sulfate (500 mg/mL) injection (3000 mg IV piggyback over 3 hours 1 time for 1 day, mixed with 100 mL of 0.9% sodium chloride. In addition, the patient received entecavir (0.5 mg oral tablet, once every 24 hours, for 9 days); acyclovir (200 mg oral tablet, 3 times a day for 9 days); doxycycline injection (100 mg IV piggyback, every 12 hours for 5 days); enoxaparin by subcutaneous injection (40 mg, injected twice per day at 6 am and 6 pm for 9 days); and ipratropium (250 µg/mL) solution for nebulization (500 µg inhalation via nebulization 4 times a day for 5 days). Additionally, the patient received 6 units of platelets due to low platelet count on the 5th day of admission; the platelet count subsequently stabilized.

Nine days later, the patient was discharged with improvement of his status. Platelet level at discharge was 118 × 109/L (normal range: 150–400 × 10^9^/L). He had a follow-up appointment with the hematology clinic 1 month later for his low platelet count. The discharge medications were valganciclovir film-coated tablet (900 mg oral BID q12h) for 30 days, as he had an elevated cytomegalovirus level of 11.687 IU/mL (normal: not detected), and apixaban (5 mg BID). The patient was then followed up at the outpatient clinic.

## 3. Discussion

A pulmonary air embolism is a rare and unintentional complication following variable procedures; it can have no or little clinical significance, or it can be lethal for patients.^[5]^ The clinical significance can be similar to that of pulmonary embolism of any etiology, leading to an elevated pulmonary artery and right ventricular pressure, which can cause right heart failure, increased ventilation/perfusion mismatch, and increased alveolar dead spaces.^[6,7]^ Large embolisms can lead to systemic hypotension and myocardial ischemia and, in worse cases, can cause arrhythmia, which may result in death.^[6,7]^

Regarding the exact definition of a large lethal air embolism in adult patients, a case report describing the accidental injection of an air embolism estimated the lethal volume to be between 200 and 300 mL or 3 to 5 mL/kg, which unfortunately led to the patient’s death.^[8]^

An air embolism during CECT is rare and, air emboli are typically small if they do occur. One study showed no association between the frequency of air embolisms, injection flow, injection site, or amount or type of contrast agent used.^[4]^

However, physicians should be informed of the presence of an air embolism, especially if it is large. The management of patients may depend on the size of the air embolism and clinical symptoms of the patient, ranging from high-flow oxygen treatment and changing the patient’s position to more drastic management such as cardiac massage and air suctioning with a large-lumen guiding catheter.^[4,9,10]^

In our case, the patient already had some shortness of breath when he presented to the emergency department, which was most likely due to a right upper lobe segmental pulmonary artery embolism. However, his worsening respiratory complaints within the first hour following CECT are likely attributable to air embolisms within the pulmonary arteries. Due to ventilation therapy, our patient developed respiratory alkalosis with secondary metabolic alkalosis, which is common and considered benign by many clinicians.^[11]^ However, studies have reported that severe alkalosis, especially in critically ill patients, has a higher mortality rate when a pH value of 7.55 is exceeded.^[12]^ Hence, awareness of the complications of both air embolisms and its treatment can lead to better patient outcomes.

## 4. Limitations

A limitation of the presented case report is the limited possibility of generalizing the study results and the difficulty of establishing a cause–effect relationship.

## 5. Conclusion

Although an air embolism is a rare complication, most air embolisms are small and cause mild symptoms; however, they can be life-threatening if large. Managing physicians should be aware of this possible complication, especially if the patient starts developing any respiratory distress or worsening symptoms shortly after CECT. Physicians should also be aware of the complications of ventilation therapy for early intervention to reduce morbidity and mortality.

## Author contributions

**Writing – review & editing:** Nora A. Altorbak.

**Conceptualization:** Rayyan A. Daghistani.

**Supervision:** Rayyan A. Daghistani, Hassan R. Al-Omaish, Thamer A. Alsaab.

**Writing – original draft:** Shahad K. Alhomaiani.
